# The Impact of Perceived Quality on Patients’ Adoption and Usage of Online Health Consultations: An Empirical Study Based on Trust Theory

**DOI:** 10.3390/healthcare13141753

**Published:** 2025-07-19

**Authors:** Shuwan Zhu, Jiahao Zhou, Nini Xu

**Affiliations:** 1 School of Management, Hefei University of Technology, Hefei 230009, China; 2023111174@mail.hfut.edu.cn (J.Z.); xunini@hfut.edu.cn (N.X.); 2 Key Laboratory of Process Optimization and Intelligent Decision-Making, Ministry of Education, Hefei 230009, China

**Keywords:** online health consultations, digital health platforms, perceived quality, trust development, online service pricing, text mining

## Abstract

**Background:** The outbreak of the COVID-19 pandemic has highlighted the importance of online health consultations, as they can help reduce the risk of contagion and infection. However, due to limited trust, these services have not yet gained widespread adoption and usage among patients. **Objective:** This research aims to examine the impact of perceived quality on patients’ adoption and usage of online health consultations from three perspectives: emotional support, responsiveness, and service continuity. Additionally, this research further explores the moderating effects of online service prices on these relationships. **Methods:** Based on trust theory, this research constructs theoretical models and empirically tests them by using a panel dataset that comprises 1255 physicians and 65,314 physician–patient communication records. **Results:** The empirical results confirm that emotional support, responsiveness, and service continuity positively influence patients’ adoption and usage behaviors. Additionally, higher online service prices negatively moderate the impact of emotional support and responsiveness on adoption behavior. Moreover, increased online service prices weaken the positive relationship between emotional support and usage behavior while strengthening the positive relationship between service continuity and usage behavior. **Conclusions:** This research extends the existing literature on online health services and provides practical guidance for platform managers, physicians, and policymakers to improve overall service acceptance.

## 1. Introduction

With the development of information and communication technology (ICT), online health consultations have attracted considerable attention. According to a recent industry report, the global digital health market was valued at approximately USD 320.17 billion in 2023 and is projected to reach USD 995.33 billion by 2032 [1]. In China, although over 27% of the population resides in the western regions, top-tier hospitals remain predominantly concentrated in the eastern metropolitan areas [2]. This geographic imbalance presents significant challenges for the Chinese government in addressing healthcare disparities stemming from the uneven distribution of high-quality medical resources [3]. As an innovative model for health service delivery, online health consultations have eliminated spatial and temporal constraints, enabling patients to communicate with physicians more efficiently [4,5,6,7]. These contextual characteristics underscore the distinctive role of online health consultations within China’s healthcare system, as they help mitigate longstanding institutional disparities. Meanwhile, the outbreak of the COVID-19 pandemic has further highlighted the importance of these services, as they can reduce the risk of contagion and infection [8]. In response, the Chinese government has actively implemented relevant policies in recent years to promote patient participation in online health consultations.

Despite these advantages, the widespread acceptance of online health consultations faces significant challenges. Previous studies have shown that older adults generally exhibit a relatively low acceptance of online health services [9,10,11]. Similarly, research by Alam et al. has indicated that even among the younger generation in Bangladesh, the acceptance rate of online health services remains significantly low [12]. Our statistical analysis of 1255 physicians in a leading online health community (April–July 2024) reveals that the ratio of the increase in total orders to the increase in total portal visits is merely 0.96%. Meanwhile, patient retention is also limited, as each physician receives an average of just seven consultations per month from patients who have previously consulted them. Given these challenges, this research focuses on strategies to promote patients’ adoption and usage of online health consultations. In this context, adoption refers to the initial online interaction with a chosen physician, while usage refers to repeated online interactions with the same physician. These two acceptance behaviors are closely related to patient acquisition and retention.

Perceived quality, defined as users’ evaluation of the excellence or superiority of the service, is a crucial factor in shaping user attitudes towards technology [13,14]. In marketing, perceived quality has been widely recognized as a predominant factor in constructing customer trust, satisfaction, and loyalty [11,15,16]. However, research by Liu and Jia has indicated that excessive emotional expression unexpectedly diminishes patients’ satisfaction with online health services [17]. Therefore, it remains uncertain whether enhanced perceived quality can promote patients’ adoption and usage of online health consultations. Consequently, this research investigates how patients’ evaluations of online health service quality influence their adoption and usage behaviors. Considering that the most critical factors in patients’ evaluations of medical service quality are the feelings of being genuinely cared for [18], this research examines the impact of perceived quality from three perspectives: emotional support, responsiveness, and service continuity.

Considering that limited trust in physicians raises potential patients’ concerns regarding the practicality of online health consultations [19], trust theory has been adopted as the theoretical foundation for analyzing patients’ decision-making processes. In the context of online health services, the development of cognitive trust and affective trust provides a comprehensive framework for understanding how perceived quality promotes patients’ adoption and usage of online health consultations. During the initial stages of impression formation within online health communities (OHCs), cognitive trust is established through rational evaluations of physicians’ profile information [20]. As physician–patient interactions progress, cognitive trust can gradually transform into affective trust, which stems from the emotional bonds between physicians and patients [21]. Specifically, this research analyzes how emotional support, responsiveness, and service continuity contribute to the development of cognitive trust and affective trust.

Unlike traditional healthcare settings, physician-driven OHCs empower physicians to set their online service prices independently [3]. Although the Chinese government actively promotes patients’ participation in online health consultations, reimbursement policies for online medical expenses remain in the pilot phase. Moreover, these policies are largely limited to internet hospitals affiliated with offline institutions [22]. As a result, patients are required to pay out of pocket for the majority of online health services. According to transaction cost economics theory, price serves as an indicator of sacrifice, influencing both demand and switching behavior [15]. Additionally, agency theory indicates that under conditions of information asymmetry and incomplete monitoring, patients (as principals) must rely on physicians (as agents) to act on their behalf. In such contexts, higher prices may heighten concerns about opportunistic behavior or misaligned incentives, thereby weakening patients’ willingness to engage with the service [23]. Accordingly, this research conceptualizes online service prices as a form of perceived risk, reflecting the potential monetary loss associated with disproportionate or ineffective services [19]. Moreover, Mayer et al. proposed that perceived risk can moderate an individual’s assessment of a trustee’s trustworthiness [24]. Therefore, this research further explores how online service prices moderate the relationships between perceived quality and acceptance behaviors. The main research questions are as follows:

Q1: How do physicians’ emotional support, responsiveness, and service continuity influence patients’ adoption of online health consultations?

Q2: How do physicians’ emotional support, responsiveness, and service continuity influence patients’ usage of online health consultations?

Q3: What are the moderating effects of online service prices on the relationships between perceived quality and acceptance behaviors?

This research collects data from 1255 physicians on Good Doctor Online (haodf.com) between April and July 2024. The application of longitudinal panel data provides valuable insights into the dynamic effects of perceived quality on patients’ adoption and usage behaviors, as well as the moderating effects of online service prices on these relationships. The empirical results confirm that emotional support, responsiveness, and service continuity positively influence patients’ adoption and usage of online health consultations. Additionally, higher online service prices negatively moderate the impact of emotional support and responsiveness on adoption behavior. Moreover, increased online service prices weaken the positive relationship between emotional support and usage behavior while strengthening the positive relationship between service continuity and usage behavior.

This research contributes to the existing literature on online health services in several ways. First, our statistical analysis, based on a large-scale panel dataset, confirms the persistence of the 1% rule within physician-driven OHCs, which has not been previously reported. This finding provides important theoretical implications for studies that focus on patients’ acceptance of online health services [25,26]. Second, this research investigates how patients evaluate the quality of online health services and how their evaluations influence their adoption and usage behaviors. While previous studies have extensively explored the complex effects of online health service quality on patients’ emotions and behaviors [17,27,28,29], few studies take a patient-centric view to examine the effectiveness of perceived quality on their acceptance behaviors, especially considering the acceptance behaviors at different stages. Third, this research clarifies the varying moderating effects of online service prices on the relationships between perceived quality and acceptance behaviors. Compared with previous studies that focus on online service pricing [15,23], this research emphases the critical role of service continuity in alleviating patients’ perceived risk of receiving disproportionate or ineffective services.

The remainder of this research is organized as follows. The next section reviews the literature on online health service quality, technology acceptance, and trust theory. Section 3 presents the hypotheses derived from the trust development perspective. The research methodologies are outlined in Section 4, including the procedures of data collection, text mining, and model construction. Section 5 presents the descriptive statistics and empirical results. The research findings are discussed in Section 6, followed by a discussion of their theoretical and practical implications. Finally, the conclusions are given in Section 7.

## 2. Literature Review and Theoretical Background

### 2.1. Online Health Service Quality

With the rapid development of ICT, online health services have transcended the spatial limitations of traditional medical care, enabling physicians and patients to communicate without time constraints [7]. As a result, extensive studies have explored the mechanisms behind online health services. From patients’ perspectives, previous studies have examined the factors affecting their satisfaction [30,31]. Zhang et al. confirmed that responses from multiple physicians within an online health community medical team can improve patient satisfaction [32]. From physicians’ perspectives, researchers have investigated factors affecting their economic and capital returns [33,34]. Yang et al. found that physicians’ use of social media and consumer sharing behavior significantly improve their online economic returns, while consumer engagement significantly improves their online capital returns [35]. Additionally, numerous studies have concentrated on factors affecting physicians’ online health service quality [36,37]. Wang et al. identified a crowding-out effect of patients’ informal payments on physicians’ intrinsic motivation to engage in physician–patient interactions [38].

Service quality refers to the overall excellence or superiority of the service [20,39]. Numerous studies have focused on measuring service quality to better understand users’ decision-making processes [39]. For example, Grönroos proposed a two-dimensional service quality model that differentiates between technical and functional quality [40]. Likewise, Parasuraman et al. developed the SERVQUAL model to assess service quality in retail and service organizations [41]. However, these generalized service quality models may not fully capture the distinctive features of online health services [22]. Therefore, context-specific models need to be customized to measure the quality of online health services.

In the field of online health services, previous studies have primarily focused on the service delivery process [28,42]. Unlike face-to-face services, online health services involve minimal physical interaction, making patients’ perceptions mainly dependent on the physicians’ behaviors [3]. Moreover, since physicians typically provide online health services through written communication, service quality can be more easily assessed and compared across different physicians [3]. Nowadays, numerous studies have explored the impact of social support on patient satisfaction [17,27]. High-quality services often incorporate social support, and enhanced social support helps improve overall service quality. Referring to related studies, emotional and informational support are the most common forms of social support encountered in peer-to-peer communities [43]. While informational support refers to the provision of medical information by physicians to help patients better understand their health conditions, emotional support is primarily reflected in their appeasing behavior towards patients [27,44]. Additionally, the influence of physicians’ voice characteristics on patient satisfaction has also been widely studied [45,46]. According to the existing literature, primary indicators for evaluating online health service quality include emotional support [43,44], informational support [17,27], and responsiveness [47,48]. Meanwhile, continuity of care, defined as ongoing communication between patients and physicians, is an essential theme in the digital transformation of health services [49]. Maintaining high service continuity is crucial for reducing irrational medical resource usage and addressing patients’ ongoing healthcare needs. Therefore, it is also a critical component of online health service quality.

### 2.2. Technology Acceptance Behaviors and Motivations

With the growing interest in the field of technology acceptance, numerous studies have increasingly focused on acceptance behaviors at different stages [10,13]. Based on the framework proposed by previous studies [10], user acceptance behaviors can be categorized into two main types: adoption and usage. Adoption refers to the initial behavior of using a technology, which occurs during the phase when an individual or organization selects a technology for the first time [10,28]. Usage denotes to the repeated behavior of using the technology during the post-adoption stage [10,13]. The active and sustained utilization of online health consultations is essential for alleviating the shortage of offline medical resources at the national level [26,50]. Therefore, investigating the strategies to promote patients’ adoption and usage of online health consultations is crucial for improving societal access to medical care.

Previous studies have extensively investigated the factors affecting user acceptance of innovative technologies [11,51,52]. Primary motivations influencing user acceptance have been identified as perceived usefulness [53,54], perceived ease of use [55,56], social influence [12,57], facilitating conditions [58,59], perceived quality [16,60], and perceived risk [61,62,63]. Among these factors, those influencing user perception are regarded as particularly significant motivations [25,64]. Notably, perceived quality, defined as the users’ evaluation of the excellence or superiority of the service, plays a critical role in constructing user trust and loyalty toward technologies [13]. Kim et al. demonstrated that perceived quality enhances user loyalty in mobile shopping applications [16]. Similarly, Cho and Kwon investigated the impact of perceived quality on service providers’ adoption and usage of service robots [14]. However, the impact of perceived quality on patients’ adoption and usage of online health consultations remains uncertain. Therefore, this research investigates how patients evaluate the quality of online health services and how their evaluations influence their adoption and usage behaviors.

This research focuses on the perceived quality of online health consultations, which is defined as patients’ evaluation of the excellence or superiority of these services [42]. While patients’ evaluations of their care often differ significantly from clinically based quality measures, they tend to make healthcare decisions based on these personal assessments [65]. Given the knowledge disparity between physicians and patients, a gap in professional understanding is inevitable during physician–patient interactions [44,66]. As a result, patients often struggle to assess the level of informational support, which is commonly measured by the amount of medical jargon used by physicians [27,65]. Meanwhile, previous studies have indicated that excessive use of medical jargon can confuse patients and even lead to dissatisfaction [17,27]. According to related studies, the most critical factors in patients’ evaluations of medical service quality are the feelings of being genuinely cared for [18]. Consequently, this research investigates the impact of perceived quality from three perspectives: emotional support, responsiveness, and service continuity.

### 2.3. Trust Theory

Trust has been widely studied in the fields of organizational behavior and marketing. It has been recognized as a fundamental element for promoting communication among unfamiliar individuals [67]. Within OHCs, limited trust in physicians raises potential users’ doubts about the practicality of online health services [19]. As a result, recent studies have increasingly adopted trust theory to explain user participation behavior in OHCs [4,48]. In the field of information systems, trust is defined as an individual’s belief in a technology’s ability to perform specific tasks. It can influence individuals’ reliance on technology [13]. Previous studies have categorized trust into different types. Yang et al. examined the effects of interpersonal and technological trust on patients’ continued usage intention of online health services [19]. McAllister delineated trust into cognitive and affective types [68]. While cognitive trust arises from rationally evaluating a trustee’s reliability, competence, and dependability, affective trust develops through emotional connections and caring behaviors. Meanwhile, Fan and Lederman investigated how cognitive and affective trust influence information adoption behaviors and contribute to relational closeness between receivers and contributors in patient-centered OHCs [21].

Previous studies have indicated that patient trust can be constructed through the information presented on physician portals [69]. Meanwhile, this trust evolves as patients participate in online health consultations [48]. This evolution can be viewed as a dynamic process of development. According to this process, trust can be categorized into initial and cumulative trust. Initial trust is typically formed when the two parties are unfamiliar with each other. Cumulative trust gradually develops through an interaction-based service process [69]. Chi et al. indicated that trust in interaction is conceptually different from trust in technology and likely encompasses a broader scope [70]. In the context of online health services, the development of cognitive and affective trust provides a comprehensive framework for understanding the transition from initial trust to cumulative trust. During the initial stages of impression formation in OHCs, cognitive trust is established through rational evaluations of physicians’ portal information [20]. As physician–patient interactions progress, cognitive trust can gradually transform into affective trust, which stems from the emotional bonds between physicians and patients [21].

**Figure 1:**
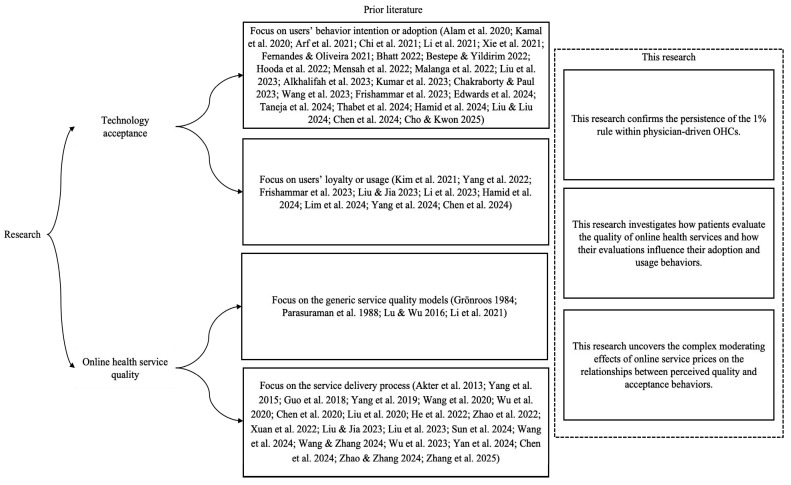
The conceptual framework [2,3,5,8,9,10,11,12,13,14,16,17,19,22,25,26,27,28,29,32,34,36,37,38,39,40,41,42,43,44,45,46,47,48,49,50,51,52,53,54,55,56,57,58,59,60,61,62,63,64,66,71].

Meanwhile, Mayer et al. proposed that perceived risk can moderate an individual’s assessment of a trustee’s trustworthiness [24]. Unlike traditional purchases, online transactions are often associated with various risks, such as receiving incorrect items and financial loss [52,61]. According to transaction cost economics theory, price serves as an indicator of sacrifice and influences both demand and switching behavior [15]. Consequently, this research conceptualizes online service prices as a form of perceived risk, representing the potential monetary loss associated with disproportionate or ineffective services [19]. To gain a deeper insight into patients’ decision-making processes, this research further explores how online service prices moderate the relationships between perceived quality and acceptance behaviors. Figure 1 shows the conceptual framework.

## 3. Hypothesis Development

This research develops several hypotheses to address the limitations of previous studies and answer the research questions. Grounded in trust theory, these hypotheses aim to examine the effects of emotional support, responsiveness, and service continuity on adoption and usage behaviors, while also exploring the moderating effects of online service prices.

### 3.1. The Effects of Physicians’ Perceived Quality on Patients’ Adoption of Online Health Consultations

Drawing on previous studies of trust development, cognitive trust is built when patients browse physicians’ portal information and assess their reliability, competence, and dependability [69]. Developing cognitive trust can directly promote patients’ adoption of online health consultations. During physician–patient interactions, a large amount of unstructured textual information is generated and displayed on physician portals [44]. These exchanged texts often contain emotional support, reflected in physicians’ comforting and reassuring behaviors toward patients. Providing more emotional support can enhance patients’ evaluations of physicians’ reliability, as it signals that physicians are not only concerned with patients’ physical health but also attentive to their psychological well-being. Therefore, this research hypothesizes the following:

**H1:**  *Physicians’ emotional support positively influences patients’ adoption of online health consultations.*

Since physicians can provide immediate responses during offline diagnoses, patients often expect the same level of efficiency in online consultations [47]. OHCs typically feature ratings of physicians’ responsiveness, which are visible on their portals. Higher responsiveness ratings can positively influence patients’ evaluations of physicians’ competence, as these ratings reflect physicians’ ability to understand and address health concerns quickly. Therefore, this research hypothesizes the following:

**H2:**  *Physicians’ responsiveness positively influences patients’ adoption of online health consultations.*

Online follow-up services enable patients to consult the same physician without returning to the hospital [39]. This service enhances the informational and interpersonal continuity between offline diagnoses and online consultations. For patients who have already experienced offline diagnoses, physicians’ encouragement to utilize online follow-up services can directly promote their adoption of online health consultations. Meanwhile, Lu et al. examined the effect of “offline-to-online” trust transfer in promoting Chinese rural residents’ utilization of online health consultations [71]. They found that such trust transfer facilitates the adoption of online health consultations among rural residents and their family members. Therefore, for patients unfamiliar with the available physicians in OHCs, the level of offline patient engagement with online follow-up services displayed on physicians’ portals can positively influence their evaluations of physicians’ dependability. It demonstrates physicians’ willingness to foster ongoing relationships and provide continuous care. Therefore, this research hypothesizes the following:

**H3:**  *Physicians’ service continuity positively influences patients’ adoption of online health consultations.*

### 3.2. The Effects of Physicians’ Perceived Quality on Patients’ Usage of Online Health Consultations

Once cognitive trust is established through patients’ rational assessments of physicians’ abilities, affective trust stems from the emotional bonds generated during physician–patient interactions [4]. Patients often encounter setbacks during prolonged treatments [72]. In some cases, the emotional toll caused by these setbacks can be more damaging than the physical condition. During these challenging moments, emotional support can reassure patients that setbacks are a normal part of the healing process and encourage them to maintain confidence and adhere to their treatments. When physicians consistently provide emotional support during interactions, it can foster a sense of being understood and deepen patients’ emotional attachment to physicians. Therefore, this research hypothesizes the following:

**H4:**  *Physicians’ emotional support positively influences patients’ usage of online health consultations.*

Additionally, physicians’ prompt responses can alleviate the uncertainty patients experience when their health concerns cannot be addressed immediately [48]. Faster reactions make patients feel respected and strengthen their emotional bonds with physicians. Therefore, this research hypothesizes the following:

**H5:**  *Physicians’ responsiveness positively influences patients’ usage of online health consultations.*

Furthermore, patients who maintain ongoing communication with specific physicians are more likely to accept the care recommended by those physicians [49]. Consequently, continuous care generally leads to better recovery outcomes. Satisfactory outcomes reinforce patients’ perceptions that their physicians are consistently concerned with the progression of their conditions. Therefore, this research hypothesizes the following:

**H6:**  *Physicians’ service continuity positively influences patients’ usage of online health consultations.*

### 3.3. The Moderating Effects of Online Service Prices on the Relationships Between Perceived Quality and Acceptance Behaviors

Consumers often encounter difficulties when comparing the quality of services offered by different sellers, particularly when available information is limited. One strategy to navigate this uncertainty is to rely on quality signals. In traditional healthcare settings, patients often use price as an indicator of quality. Due to limited medical knowledge, they tend to associate higher fees with better service, leading them to select physicians who charge more [3]. However, OHCs provide patients with abundant information, helping them identify substandard physicians and make more informed decisions [73,74]. Meanwhile, online health services are considered a complement to traditional hospital care, primarily addressing non-emergency medical needs. According to transaction cost economics theory, consumers interpret online service prices as a signal of sacrifice, influencing their purchasing and switching decisions [15,61,63]. Consequently, this research conceptualizes online service prices as a form of perceived risk, representing the potential monetary loss associated with disproportionate or ineffective services [15]. Moreover, Mayer et al. proposed that an individual’s assessment of a trustee’s trustworthiness can be moderated by the perceived risk [24]. In the field of technology acceptance, Kumar et al. confirmed the negatively moderating effect of perceived risk on the relationship between users’ behavioral intentions and their actual use of mobile banking services [62]. Therefore, this research hypothesizes the following:

**H7a:**  *Online service prices negatively moderate the relationship between physicians’ emotional support and patients’ adoption of online health consultations.*

**H7b:**  *Online service prices negatively moderate the relationship between physicians’ responsiveness and patients’ adoption of online health consultations.*

**H7c:**  *Online service prices negatively moderate the relationship between physicians’ service continuity and patients’ adoption of online health consultations.*

From an economic perspective, price not only serves as an indicator of demand but also provides valuable information about supply dynamics [15]. Physicians often have heavy workloads in physical hospitals, leaving them limited time to dedicate to OHCs. The pricing mechanisms within OHCs enable physicians to manage demand by adjusting service prices. When physicians raise online service prices, it may signal potential risks of providing inefficient online services. Consequently, patients tend to focus more on service continuity rather than emotional support and responsiveness. Service continuity represents physicians’ commitment to maintaining ongoing relationships with patients. It reassures patients that their healthcare needs will be consistently addressed, regardless of physicians’ immediate availability. This sense of continuity helps mitigate concerns about potential service disruptions and fosters a sense of security in the patient–physician relationship. Therefore, this research hypothesizes the following:

**H8a:**  *Online service prices negatively moderate the relationship between emotional support and patients’ usage of online health consultations.*

**H8b:**  *Online service prices negatively moderate the relationship between responsiveness and patients’ usage of online health consultations.*

**H8c:**  *Online service prices positively moderate the relationship between service continuity and patients’ usage of online health consultations.*

**Figure 2:**
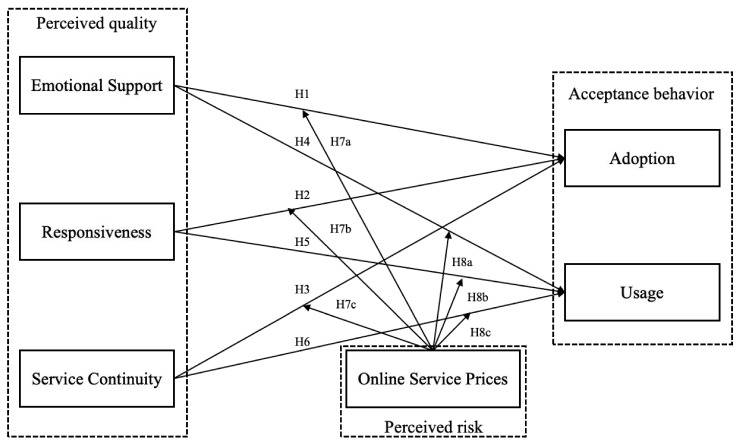
The conceptual model.

The conceptual model guiding this research is illustrated in Figure 2.

## 4. Research Methodology

This section outlines the research context, data collection process, variable measurement, text mining procedure, and the construction of empirical models.

### 4.1. Research Context and Data Collection

This research focuses on Good Doctor Online (haodf.com), one of the most professional physician-driven OHCs in China. It provides an ideal context for testing hypotheses. First, the platform positions online health services as the core of its business. It offers a variety of services, including written consultations, telephone consultations, video consultations, and online follow-up services. Second, this platform makes physician information easily accessible. It significantly narrows the information gap between physicians and patients. Third, this platform displays detailed and specific physician–patient interactions on physician portals. By leveraging this platform, this research gathers background information and service behaviors of physicians to build the dataset.

A Python 3.10-based web crawler was developed to collect the research data from the platform. To ensure content dependability, this research selected six representative diseases: coronary heart disease, cerebral infarction, lung cancer, stomach cancer, diabetes, and asthma. According to the 2022 China Health Statistics Yearbook [75], the mortality rates for these diseases (per 100,000 population) in descending order are as follows: coronary heart disease (283.27), cerebral infarction (107.78), lung cancer (96.87), diabetes (38.89), stomach cancer (35.76), and asthma (1.72). This selection encompassed a wide range of mortality rates, reflecting the diversity and representativeness of the diseases included in this research. To select physicians for each disease, this research initially collected 60 physicians listed on the classification page for each province. When the number of available physicians was insufficient, all physicians were included to ensure a comprehensive sample of active physicians on the platform. After excluding physicians who did not offer written consultation and whose last online activity occurred more than one month prior, this research obtained a sample of 1255 physicians.

**Figure 3:**
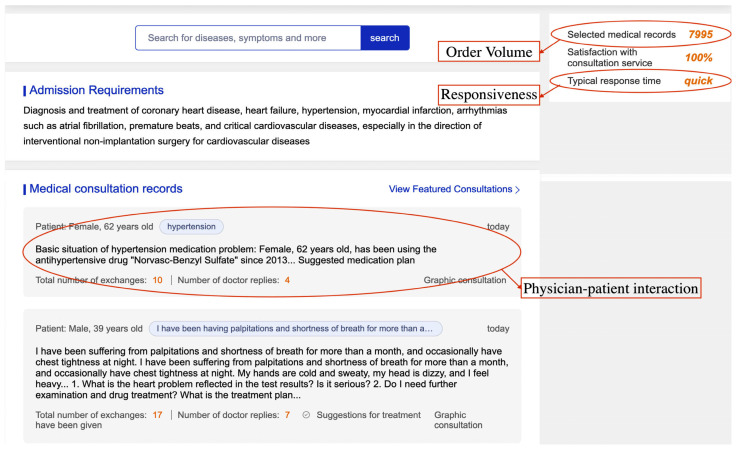
An example of a physician’s online health consultation page.

Data was collected four times between April and July 2024, resulting in a panel dataset containing three months of information. All structured data collection was completed within a single day to ensure accuracy and consistency in variable measurement. The last response time was used as the criterion to retrieve all physician–patient interactions where physicians had responded within the past month. After excluding invalid interactions with fewer than three rounds, as well as telephone or video consultations for which the full content could not be viewed as text, this research obtained 65,314 valid physician–patient interactions containing 641,664 physicians’ responses. An example of a physician’s online health consultation page on Good Doctor Online is presented in Figure 3.

### 4.2. Variable Measurement

#### 4.2.1. Dependent Variables

The dependent variables in this research include adoption $\left({Adoption}_{it}\right)$ and usage $\left({Usage}_{it}\right)$. Adoption refers to the behavior of consulting with a chosen physician for the first time [10]. This research uses the increase in patients as a proxy to measure this variable. Usage refers to the behavior of consulting with the same physician repeatedly [10]. It is measured by the increase in orders minus the increase in patients.

#### 4.2.2. Independent Variables

The independent variables in this research include emotional support $\left({Emotional\;Support}_{it}\right)$, responsiveness (${Responsiveness}_{it})$, and service continuity $\left({Service\;Continuity}_{it}\right)$.

Emotional support refers to the positive emotional reinforcement physicians provide during physician–patient interactions [28]. To quantify the extent of emotional support, this research calculates the ratio of the number of sentences containing emotional support to the number of valid physician–patient interactions. The data is extracted from the text of physician–patient interactions by using a text mining technique.

Responsiveness refers to the ability of providers to address patients’ needs promptly [65]. This research measures physician responsiveness by encoding the average response time, which is rated by the platform algorithm. Physicians’ responsiveness is coded as follows: a value of 1 is assigned if the rating is “None”, a value of 2 is assigned if the rating is “Slow” or “Slower”, and a value of 3 is assigned if the rating is “Normal”, “Fast”, or “Faster”.

Service continuity refers to ongoing communication between physicians and patients [49]. Online follow-up services enable patients to stay in touch with the same physicians through OHCs [39]. To measure service continuity, this research uses the ratio of the increase in online follow-up patients to the increase in total patients. This ratio not only demonstrates the extent to which physicians encourage patients to utilize online follow-up services after offline treatments but also reflects their willingness to foster ongoing relationships and provide continuous care.

#### 4.2.3. Moderator Variable

Since written consultation is the most common form of consultation offered by physicians on the platform, this research uses the prices of written consultation as the measure for physicians’ online service prices $\left({Price}_{it}\right)$.

#### 4.2.4. Control Variables

Considering that higher recommendation heat displayed on a physician portal indicates greater popularity, patients may perceive physicians with higher recommendation heat as more likely to provide thoughtful online health consultations. Therefore, this research adopts the recommendation heat (*Recommendation_it_*) as the first control variable to examine the impact of perceived quality on adoption behavior. To better explore these effects, this research also introduces the increase in physicians’ articles $\left({Article}_{it}\right)$ as another control variable into the models. Additionally, this research incorporates the increase in gifts $\left({Gift}_{it}\right)$ as the control variable to enhance the analysis of the impact of perceived quality on usage behavior. Gift-giving is a voluntary behavior undertaken by patients following their interactions with physicians, typically reflecting appreciation for the care they have received. As such, this variable can be used as a proxy for physicians’ historical service performance. It serves to capture residual variation in quality-related attributes that are not directly accounted for by the specific variables measured in this research [44,47]. Table 1 presents the details regarding the measurements of all variables.

### 4.3. The Procedure of Text Mining

**Figure 4:**
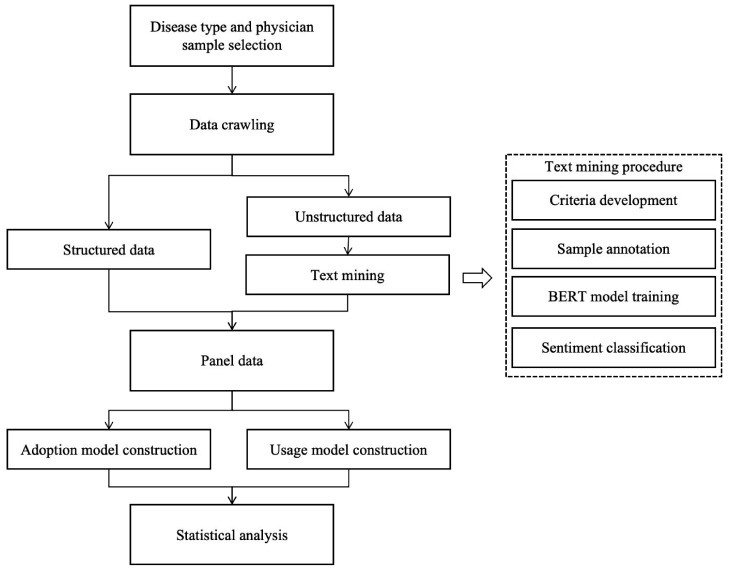
Overview of research procedures.

Since physician replies typically lack negative emotions, the assessment focused on identifying whether the sentences contained emotional support. This research randomly selected 4000 physician replies from each disease category, creating a dataset of 24,000 samples. Based on prior studies [43,44], this research established several criteria to label the samples: (1) daily greetings, (2) positive comments on recovery outcomes (e.g., “quite a bit of improvement”), (3) reassurance and encouragement (e.g., “don’t worry” and “we’ll work together”), and (4) clarifications aimed at alleviating uncertainty regarding treatment modalities and recovery processes (e.g., “the success rate is quite high” and “it will improve over time”). Sentences meeting these criteria were tagged as 1, while all other sentences were tagged as 0. The labeling criteria and examples are summarized in Appendix A. After annotating the samples, this research randomly selected 80% of positive- and non-positive-sentiment sentences to form the training set, reserving the remaining 20% for the test set. Since positive-sentiment sentences accounted for only about 25% of the total, this approach ensured that the classifier effectively learned the characteristics of sentences containing emotional support. The training set was then used to fine-tune the BERT model. After tuning, the following parameters were set as follows: maximum sequence length of 128, training batch size of 16, learning rate of 2 × 10^−5^, and 10 training epochs. The performance of the fine-tuned BERT model on the test set was as follows: Accuracy of 94.5%, AUC of 91.0%, precision of 90.0%, recall of 91.0%, F1-score of 90.5%, and loss of 23.0%. Finally, the fine-tuned BERT model was used to classify the remaining sentences. Figure 4 provides an overview of the research procedures.

### 4.4. Model Construction

This research constructs four empirical models to test our hypotheses. To mitigate the skewness of the variables and account for the presence of zeros in the data [76], the natural logarithm transformation plus one is performed on the following variables: ${Adoption}_{it}$, ${Usage}_{it}$, ${Emotional\;Support}_{it}$, ${Price}_{it}$, ${Article}_{it}$, and ${Gift}_{it}$. For clarity and consistency, this research retains the original variable names to denote the log-transformed versions of these variables. The multiple regression models are as follows:

(1) $${Adoption}_{it}=\beta_{0}+\beta_{1}{Emotional\;Support}_{it}+\beta_{2}{Responsiveness}_{it}\;\;\;\;\;\;\;\;\;\;\;\;\;\;\;\;\;\;\;\;\;\;\;\;\;\;\;\;\;\;\;\;+\beta_{3}{Service\;Continuity}_{it}+\beta_{4}{Recommendation}_{it}\;+\beta_{5}{Article}_{it}+\alpha_{i}+\gamma_{t}+\varepsilon_{it}$$

(2) $${Adoption}_{it}=\;\beta_{0}+\;\beta_{1}\;{Emotional\;Support}_{it}+\;\beta_{2}\;{Responsiveness}_{it}\;+\beta_{3}\;{Service\;Continuity}_{it}\;\;\;\;\;\;\;\;\;\;\;\;\;\;\;\;+\beta_{4}\;{Emotional\;Support}_{it}\times \;{Price}_{it}\;\;+\;\beta_{5}\;{Responsiveness}_{it}\times \;{Price}_{it}\;\;\;\;\;\;\;\;+\;\beta_{6}\;{Service\;Continuity}_{it}\times \;{Price}_{it}\;\;\;\;\;\;\;\;\;\;\;\;\;\;\;\;\;\;\;\;\;\;\;\;\;\;\;\;\;\;\;\;\;\;\;\;\;\;\;\;+\;\beta_{7}\;{Recommendation}_{it}+\;\beta_{8}\;{Article}_{it}+\;\alpha_{i}+\;\gamma_{t}+\;\varepsilon_{it}$$

(3) $${Usage}_{it}=\beta_{0}+\beta_{1}{Emotional\;Support}_{it}+\beta_{2}{Responsiveness}_{it}\;\;\;\;\;\;\;\;\;\;\;\;\;\;\;\;\;\;\;\;\;\;\;\;\;\;\;\;\;\;\;\;\;+\beta_{3}{Service\;Continuity}_{it}+\beta_{4}{Gift}_{it}+\alpha_{i}+\gamma_{t}+\varepsilon_{it}$$

(4) $${Usage}_{it}=\beta_{0}+\beta_{1}{Emotional\;Support}_{it}+\beta_{2}{Responsiveness}_{it}\;\;+\beta_{3}Service\;{Continuity}_{it}\;\;\;\;\;\;\;\;\;\;\;\;\;\;\;+\beta_{4}{Emotional\;Support}_{it}\times {Price}_{it}\;+\beta_{5}{Responsiveness}_{it}\times {Price}_{it}\;\;\;\;\;\;\;\;\;\;\;\;\;\;\;\;\;\;\;\;\;\;\;\;\;\;\;\;\;\;\;\;\;\;\;\;\;\;\;\;+\beta_{6}{Service\;Continuity}_{it}\times {Price}_{it}+\beta_{7}{Gift}_{it}+\alpha_{i}+\gamma_{t}\;+\varepsilon_{it}\;\;\;\;\;\;\;\;\;\;\;\;\;\;\;\;\;\;\;\;\;\;\;\;\;\;\;\;\;\;\;\;\;\;\;\;\;\;\;\;\;$$

Here, i denotes the physician, and t denotes the month. $\alpha_{i}$ refers to the physician-fixed effects, while $\gamma_{t}$ represents the month-fixed effects. The physician-fixed and month-fixed effects absorb unobservable factors related to individuals and time that remain constant. This approach helps mitigate the endogeneity concerns in research models.

Finally, this research uses the variance inflation factor (VIF) approach to assess multicollinearity in the research models. The results presented in Table 2 show that the VIFs for all variables are below the widely accepted threshold (VIF < 10), suggesting that multicollinearity is not a significant concern in the research models.

## 5. Result

This section starts with descriptive statistics of the collected panel data and the correlations among the research variables, followed by the empirical results and robustness checks.

### 5.1. Descriptive Statistics and Correlations

This research uses the STATA software (version 18.0) to analyze the data and test the hypotheses. The dataset provides an overview of online health consultations conducted by 1255 physicians across six diseases over a three-month period. The research data reveals that the ratio of total increases in orders to total increases in portal visits is merely 0.96%, indicating that the “1% rule” also applies within physician-driven OHCs. The 1% rule posits that 90% of participants observe content without contributing, 9% contribute sparingly, and 1% generate the majority of new content [77]. This framework provides a valuable perspective for understanding online community participation patterns and the network effects they generate.

**Figure 5:**
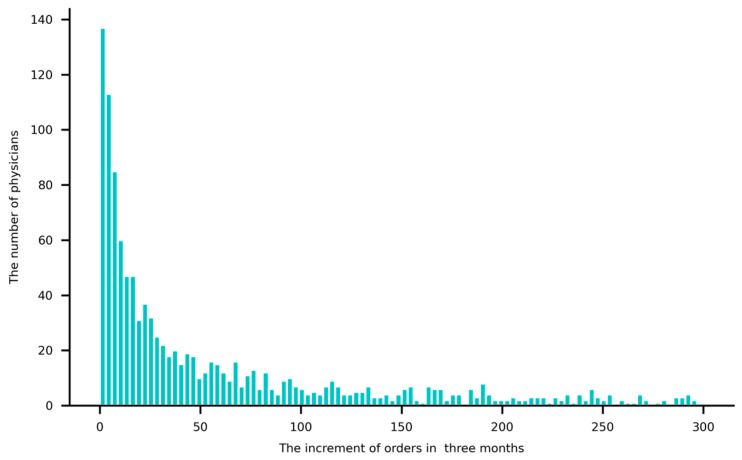
The distribution of order increases over the three months.

Figure 5 illustrates the distribution of order increases over the three months, excluding samples where increases exceeded 300. The skewed distribution reveals that more than half of the physicians received fewer than 50 orders during this period. Notably, 46 physicians did not receive any orders, despite remaining active on the platform.

**Figure 6:**
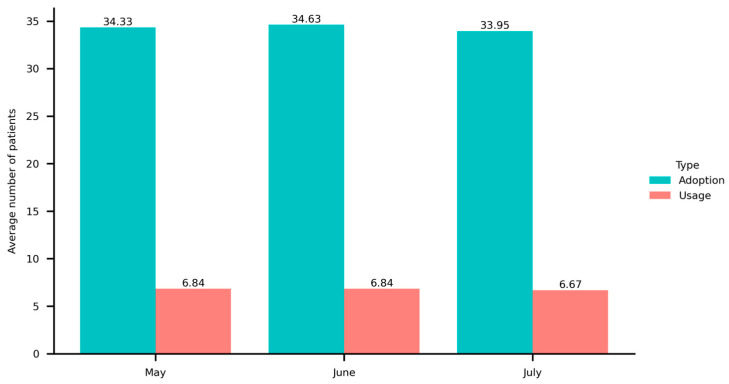
The average number of patients treated by physicians in three months.

Furthermore, Figure 6 displays the average number of patients treated by physicians, categorized into adoption and usage groups. The data shows that each physician, on average, received approximately seven consultations per month from patients who had previously consulted them. These findings indicate that online health consultations have not yet gained widespread acceptance among patients. They mainly use the platform to access information from physicians’ portals.

The descriptive statistics and correlations for the research variables are presented in Appendix B. The results indicate that the independent variables are significantly correlated with the dependent variables, and the correlations between different variables stay below 0.8, indicating no problems with multicollinearity.

### 5.2. Empirical Results

Table 3 presents the results of models 1 and 2, estimated by ordinary least squares (OLS). The models are presented hierarchically: Column 1 includes the model with control and moderator variables, while columns 2 and 3 introduce the independent variables and interaction terms. The results in column 2 of Table 3 show that emotional support $\left(\beta_{1}=0.239,\;\;p<0.01\right)$, responsiveness $\left(\beta_{2}=0.111,\;\;p<0.01\right)$, and service continuity $\left(\beta_{3}=0.269,\;\;p<0.01\right)$ have significant and positive effects on adoption behavior. Thus, hypotheses H1, H2, and H3 are supported. This demonstrates that the information displayed on physicians’ portals regarding perceived quality significantly influences patients’ initial adoption of online health consultations. The results in column 3 further reveal that higher online service prices negatively moderate the relationship between emotional support and adoption behavior $\left(\beta_{4}=-0.132,\;\;p<0.1\right)$. Additionally, the interaction between online service prices and responsiveness shows a significant and negative coefficient $\left(\beta_{5}=-0.051,\;\;p<0.01\right)$. This suggests that higher service prices weaken the relationship between responsiveness and adoption behavior. Therefore, H7a and H7b are supported. However, the coefficient of the interaction between online service prices and service continuity is not significant $\left(\beta_{6}=0.013,\;\;p>0.1\right)$, indicating that H7c is not supported.

Because usage data contains many zero values, this research employs Poisson Pseudo Maximum Likelihood with Multiple High-dimensional Fixed Effects (PPMLHDFE) to estimate models 3 and 4. The models are similarly presented in hierarchical form in Table 4. The results in column 2 of Table 4 show that emotional support $\left(\beta_{1}=0.389,\;\;p<0.01\right)$, responsiveness $\left(\beta_{2}=0.372,\;\;p<0.01\right)$, and service continuity $\left(\beta_{3}=0.216,\;\;p<0.01\right)$ significantly and positively influence usage behavior. Hence, hypotheses H4, H5, and H6 are supported. This indicates that the service quality patients perceive during physician–patient interactions significantly promotes their continued usage of online health consultations. According to the results in column 3, increased online service prices negatively moderate the relationship between emotional support and usage behavior $\left(\beta_{4}=-0.786,\;\;p<0.01\right)$. Hypothesis H8a is supported. In contrast, the coefficient of the interaction between online service prices and responsiveness is not significant $\left(\beta_{5}=-0.048,\;\;p>0.1\right)$. This leads to the rejection of hypothesis H8b. Corresponding to hypothesis H8c, the interaction between online service prices and service continuity has a significant and positive coefficient $\left(\beta_{6}=0.261,\;\;p<0.05\right)$. This indicates that increased online service prices strengthen the relationship between service continuity and usage behavior.

### 5.3. Robustness Check

This research employs several methods to validate the robustness of the empirical results. First, this research conducts a robustness check on outliers to verify the validity of findings on adoption behavior. Specifically, this research filters out extreme values by retaining samples that fall between the 1st and 99th percentiles of the Adoption variable. Second, this research randomly selects approximately 90% of the physicians as a subsample to re-estimate the empirical models. The detailed results are presented in Table 5. They remain consistent with those reported in Table 3, further validating the robustness of our findings on adoption behavior.

Furthermore, this research re-estimates the empirical models by standardizing the Usage variable. Additionally, to address potential concerns about applying regression models to highly skewed data with an excessive number of zero values, this research employs an untransformed Usage variable and applies a Poisson fixed-effects model as an additional robustness check. The detailed results, presented in Table 6, consistently validate the robustness of our findings on usage behavior.

Finally, this research examines potential disease-level heterogeneity, which may influence patients’ acceptance behaviors. To address this issue, this research collects mortality rates for individual diseases included in the sample. Additional regressions are conducted by incorporating disease-fixed effects to control for variations across the six representative diseases. The results, presented in Table 7 and Table 8, are consistent with the prior empirical results, thereby further enhancing the validity of our findings.

## 6. Discussion and Implications

### 6.1. Discussion

Online health consultations provide a new avenue for patients to interact with physicians. Unlike Western cultures, Chinese culture is deeply rooted in collectivism, making interpersonal trust essential in social interactions. Based on trust theory, this research examines the impact of perceived quality on patients’ acceptance behaviors in the context of online health consultations. Additionally, this research also explores the moderating effects of online service prices on the relationships between perceived quality and acceptance behaviors. Drawing on data from a leading online health community in China, this research develops several empirical models to test the hypotheses and derive several insightful findings. Firstly, the empirical results confirm that emotional support, responsiveness, and service continuity positively influence patients’ adoption of online health consultations. When patients browse physicians’ portals, information about these factors significantly shapes their evaluations of physicians’ reliability, competence, and dependability [69]. These rational assessments help foster the construction of cognitive trust, which directly promotes their adoption of online health consultations [48].

Secondly, the results indicate that emotional support, responsiveness, and service continuity also positively influence patients’ usage of online health consultations. Enhanced emotional support and shorter response times during physician–patient interactions strengthen patients’ sense of understanding and respect. Furthermore, enhanced service continuity enables patients to achieve better health outcomes and reinforces their perception of physicians’ continuous care. These positive experiences help transform cognitive trust into affective trust, thereby promoting their usage of online health consultations [48].

Thirdly, this research clarifies how online service prices moderate the relationships between perceived quality and acceptance behaviors. For patients who have not previously interacted with physicians in OHCs, higher online service prices negatively moderate the relationships between emotional support, responsiveness, and adoption behavior. Higher online service prices heighten patients’ concerns about potential monetary loss, which in turn reduces the importance they place on emotional support and responsiveness when making adoption decisions. However, the moderating effect of online service prices on the relationship between service continuity and adoption behavior is not statistically significant. As a convenient channel for patients to maintain communication with their physicians, their utilization of online follow-up services can be regarded as an extension of offline recommendations. Within the context of OHCs, Wang et al. demonstrated that offline recommendations exert a greater influence on enhancing patient satisfaction compared to online recommendations [30]. Similarly, Lu et al. emphasized the critical role of trust transfer from offline to online settings in facilitating the adoption of online health consultations among rural residents and their families [71]. Accordingly, it is reasonable to infer that service continuity can more effectively alleviate patients’ perceived risk of receiving disproportionate care than emotional support and responsiveness.

Finally, online service prices exhibit different moderating effects for patients who have prior interactions with physicians on OHCs. Specifically, increased online service prices weaken the positive relationship between emotional support and usage behavior while strengthening the positive relationship between service continuity and usage behavior. When physicians raise their online service prices, it may signal a reduced likelihood of providing effective online care. This is often due to heavy workloads in their offline clinical settings [15]. In such instances, the decision-making logic of experienced patients may change. Previous studies have indicated that mutual understanding between physicians and patients encourages a greater focus on treatment outcomes [48]. Under outcome-oriented evaluation criteria, the benefits of emotional support and responsiveness may become less discernible [44,48]. Nevertheless, compared to emotional support, responsiveness plays a more critical role in addressing urgent medical needs [47]. This distinction helps explain why increased online service prices exert a stronger negative moderating effect on the relationship between emotional support and usage behavior than on the relationship between responsiveness and usage behavior. Meanwhile, the positive moderating effect of increased online service prices on the relationship between service continuity and usage behavior has been empirically confirmed. Given the essential role of service continuity in alleviating patients’ perceived risk regarding service effectiveness, this finding is consistent with previous studies indicating that consumers are more willing to pay premium prices to providers who can reduce perceived risk and induce trust [15].

### 6.2. Theoretical Implications

This research offers several critical theoretical contributions. First, our statistical analysis confirms the persistence of the 1% rule within physician-driven OHCs. This indicates that online health consultations have not yet gained widespread acceptance among patients. This finding provides important theoretical implications for studies focusing on patients’ acceptance of online health services [25,26]. Concentrating on this issue, this research extends the application of trust theory to the context of online health services by elucidating the mechanisms of how perceived quality influences patients’ acceptance behaviors.

Second, unlike previous studies that focus on the complex effects of online health service quality on patients’ emotions and behaviors [17,27,28,29], this research adopts a patient-centric approach to examine the impact of perceived quality on their acceptance behaviors, especially considering the acceptance behaviors at different stages. Based on patients’ evaluations of online health service quality, this research measures the perceived quality from three perspectives: emotional support, responsiveness, and service continuity. The empirical results validate the mechanisms by which emotional support, responsiveness, and service continuity influence patients’ adoption and usage of online health consultations.

Third, this research clarifies the varying moderating effects of online service prices on the relationships between perceived quality and acceptance behaviors. The empirical results indicate that higher online service prices negatively moderate the relationships between emotional support, responsiveness, and adoption behavior. Furthermore, increased online service prices weaken the effect of emotional support while strengthening the effect of service continuity on usage behavior. These insights enrich the theoretical foundations of research on online service pricing, particularly in understanding the intricate interplay among perceived quality, online service prices, and acceptance behaviors [15,23].

### 6.3. Practical Implications

This research also offers several practical implications for platform managers, physicians, and policymakers. First, due to the persistently low levels of service adoption, online health platforms should prioritize strategies that promote greater patient participation to fully realize the potential benefits of these services. Specifically, platform managers should focus on providing more transparent and comprehensive information to alleviate the cognitive burden that patients experience when selecting physicians. As generative artificial intelligence (AI) technologies continue to reshape global models of care, intelligent customer service agents have been increasingly deployed in OHCs to recommend appropriate physicians [78,79]. This trend highlights the growing integration of AI into patient decision-making processes. Based on our findings, the development of more user-friendly intelligent customer service agents is likely to further promote patients’ adoption of online health consultations.

Second, this research helps physicians understand how patients evaluate the quality of online health services and the roles that emotional support, responsiveness, and service continuity play in promoting patients’ usage of online health consultations. From patients’ perspectives, the most critical factors influencing their perception of medical service quality are the feelings of being genuinely cared for. Therefore, physicians should focus on offering more emotional support, responding to patients promptly, and providing continuous care to retain online patients.

Third, this research recommends that government health authorities should develop more comprehensive regulatory frameworks to address patients’ concerns about potential opportunistic behaviors in OHCs. In practice, implementing more timely complaint and feedback mechanisms can facilitate more rigorous regulation of online service pricing, thereby further mitigating the persistent information asymmetry in the healthcare market. Additionally, policies related to health insurance reimbursement for online health services should be piloted more widely and scaled up more rapidly to promote greater patient participation.

### 6.4. Limitations and Future Research

Although this research provides a comprehensive analysis of the research questions, it still has several limitations and presents opportunities for further investigation. First, due to data availability constraints, the research dataset only contains physician-level characteristics. However, patient demographics, such as disease severity and family opinions, may influence patients’ decisions regarding online health consultations. In addition, external factors, such as advertising and social recommendations, may also shape patients’ perception of service quality. Future research could incorporate these factors as control variables to better address potential endogeneity issues and account for unobserved external influences.

Second, although this research incorporates online service prices as a moderating variable, it is treated in a relatively general manner. Our analysis does not consider patients’ price sensitivities and specific price thresholds. These factors may affect how patients respond to online service pricing in the context of OHCs. Future research can delve deeper into these aspects to provide a more nuanced understanding of online service pricing.

Third, the dataset used to test the hypotheses is derived from a single physician-driven online health community in China. Although the dataset is representative, the findings may be specific to this platform. In various cultural contexts, constructs such as emotional support and price-related risk perception may differ significantly across healthcare systems that emphasize patient-centered care or offer more comprehensive public insurance coverage. Therefore, future research can utilize data from other prominent OHCs in countries with diverse healthcare systems or varying degrees of digital health integration to enhance the generalizability of our findings.

## 7. Conclusions

Online health consultations overcome the spatial limitations of traditional medical care, enabling physicians and patients to communicate without time constraints [7,32]. However, these services have not yet been widely accepted by patients. Therefore, this research investigates how perceived quality influences patients’ adoption and usage of online health consultations. Based on trust theory, this research examines the impact of perceived quality from three perspectives: emotional support, responsiveness, and service continuity. The empirical results confirm that emotional support, responsiveness, and service continuity positively influence their adoption and usage behaviors. Furthermore, this research clarifies the varying moderating effects of online service prices on the relationships between perceived quality and acceptance behaviors. Overall, this research makes valuable contributions to the theoretical understanding and practical applications of online health services.

## Figures and Tables

**Table 1:** Variable descriptions.

Variable		Description	Measurement
Dependent variable	${Adoption}_{it}$	The behavior of consulting with a chosen physician for the first time.	The increase in patients of physician i in month t.
	${Usage}_{it}$	The behavior of consulting with the same physician continuously and repeatedly.	The difference between the increase in orders and the increase in patients for physician i in month t.
Independent variable	${Emotional\;Support}_{it}$	The positive emotional reinforcement provided by physicians during their interactions with patients.	The ratio of the number of sentences containing emotional support to the number of valid physician–patient interactions of physician i in month t.
	${Responsiveness}_{it}$	The ability of providers to address patients’ needs promptly.	The average response time, as rated by Good Doctor Online, of physician i in month t.
	${Service\;Continuity}_{it}$	The extent to which physicians encourage patients to engage with online follow-up services after offline treatments.	The ratio of the increase in online follow-up patients to the increase in total patients of physician i in month t.
Moderator variable	${Price}_{it}$	The prices of online health services set by physicians.	The price of written consultations of physician i in month t.
Control variable	${Recommendation}_{it}$	The recommendation heat displayed on physician portals.	The recommendation heat of physician i in month t.
	${Article}_{it}$	The articles written by physicians and posted on portals.	The increase in articles of physician i in month t.
	${Gift}_{it}$	A voluntary behavior that patients engage in after interactions with physicians.	The increase in gifts of physician i in month t.

**Table 2:** The results of VIF values.

Variable	Adoption		Usage	
	VIF	1/VIF	VIF	1/VIF
Emotional Support	1.26	0.7920	1.28	0.7841
Responsiveness	1.34	0.7471	1.34	0.7447
Service Continuity	1.24	0.8079	1.10	0.9062
Price	1.14	0.8782	1.08	0.9288
Recommendation	1.37	0.7286		
Article	1.05	0.9568		
Gift			1.17	0.8573
Mean VIF	1.23		1.19	

**Table 3:** Estimation results of adoption behavior.

Variable	(1)	(2)	(3)
Constant	1.166 ***	0.651 ***	0.380
	(4.82)	(2.96)	(1.63)
Recommendation	0.029	0.036	0.038
	(0.68)	(0.93)	(0.97)
Article	0.072 **	0.074 **	0.073 **
	(2.07)	(2.34)	(2.31)
Price	−0.139	−0.116	0.036
	(−1.55)	(−1.43)	(0.39)
Emotional Support		0.239 ***	0.488 ***
		(6.78)	(3.44)
Responsiveness		0.111 ***	0.201 ***
		(14.14)	(6.45)
Service Continuity		0.269 ***	0.244 ***
		(13.90)	(3.26)
Price × Emotional Support			−0.132 *
			(−1.80)
Price × Responsiveness			−0.051 ***
			(−3.02)
Price × Service Continuity			0.013
			(0.31)
Physician-fixed effects	Yes	Yes	Yes
Month-fixed effects	Yes	Yes	Yes
Observations	3765	3765	3765
F	2.59	96.31	66.27
Prob > F	0.052	0.000	0.000
R-squared	0.950	0.959	0.959
Adjusted R-squared	0.924	0.938	0.939

Notes: *t* statistics in parentheses; * *p* < 0.10, ** *p* < 0.05, *** *p* < 0.01.

**Table 4:** Estimation results of usage behavior.

Variable	(1)	(2)	(3)
Constant	1.004 **	−0.298	−0.626
	(2.33)	(−0.65)	(−0.98)
Gift	0.141 ***	0.123 ***	0.124 ***
	(4.86)	(4.26)	(4.28)
Price	−0.630 ***	−0.613 ***	−0.436
	(−2.87)	(−2.77)	(−1.34)
Emotional Support		0.389 ***	1.888 ***
		(2.88)	(3.44)
Responsiveness		0.372 ***	0.463 ***
		(9.00)	(3.11)
Service Continuity		0.216 ***	−0.268
		(3.61)	(−1.25)
Price × Emotional Support			−0.786 ***
			(−2.82)
Price × Responsiveness			−0.048
			(−0.65)
Price × Service Continuity			0.261 **
			(2.29)
Physician-fixed effects	Yes	Yes	Yes
Month-fixed effects	Yes	Yes	Yes
Observations	2955	2955	2955
Log pseudolikelihood	−2182.692	−2176.316	−2175.778
Wald chi2	34.15	137.96	147.27
Prob > chi2	0.000	0.000	0.000
Pseudo R2	0.195	0.197	0.197

Notes: z statistics in parentheses; * *p* < 0.10, ** *p* < 0.05, *** *p* < 0.01.

**Table 5:** Robust check for adoption behaviors.

Variable	Removing Outliers			Subsample Regression		
	(1)	(2)	(3)	(4)	(5)	(6)
Constant	1.063 ***	0.551 **	0.279	1.227 ***	0.683 ***	0.380
	(4.47)	(2.55)	(1.22)	(4.83)	(2.96)	(1.55)
Recommendation	0.027	0.034	0.035	0.021	0.031	0.032
	(0.63)	(0.88)	(0.92)	(0.49)	(0.80)	(0.82)
Article	0.072 **	0.074 **	0.073 **	0.039	0.046	0.044
	(2.11)	(2.38)	(2.35)	(1.02)	(1.31)	(1.28)
Price	−0.094	−0.071	0.082	−0.154	−0.127	0.048
	(−1.06)	(−0.89)	(0.90)	(−1.58)	(−1.45)	(0.47)
Emotional Support		0.239 ***	0.492 ***		0.264 ***	0.580 ***
		(6.90)	(3.53)		(6.82)	(3.77)
Responsiveness		0.110 ***	0.199 ***		0.113 ***	0.214 ***
		(14.38)	(6.50)		(13.19)	(6.24)
Service Continuity		0.262 ***	0.256 ***		0.266 ***	0.216 **
		(13.78)	(3.48)		(12.29)	(2.53)
Price × Emotional Support			−0.134 *			−0.169 **
			(−1.86)			(−2.11)
Price × Responsiveness			−0.050 ***			−0.058 ***
			(−3.01)			(−3.08)
Price × Service Continuity			0.002			0.026
			(0.05)			(0.54)
Physician-fixed effects	Yes	Yes	Yes	Yes	Yes	Yes
Month-fixed effects	Yes	Yes	Yes	Yes	Yes	Yes
Observations	3765	3765	3765	3299	3299	3299
F	2.13	97.40	67.07	1.35	80.42	55.87
Prob > F	0.094	0.000	0.000	0.255	0.000	0.000
R-squared	0.944	0.955	0.955	0.952	0.961	0.962
Adjusted R-squared	0.916	0.932	0.932	0.925	0.939	0.939

Notes: *t* statistics in parentheses; * *p* < 0.10, ** *p* < 0.05, *** *p* < 0.01.

**Table 6:** Robust check for usage behaviors.

Variable	Standardized *Usage* Variable			Poisson Fixed-Effects Model		
	(1)	(2)	(3)	(4)	(5)	(6)
Constant	−1.186 **	−2.454 ***	−3.004 ***	4.226 ***	3.098 ***	2.537 ***
	(−2.29)	(−4.39)	(−3.63)	(8.26)	(5.81)	(3.46)
Gift	0.224 ***	0.220 ***	0.228 ***	0.224 ***	0.220 ***	0.228 ***
	(4.22)	(4.42)	(4.54)	(6.64)	(6.49)	(6.69)
Price	−0.630 **	−0.630 **	−0.358	−0.630 ***	−0.630 ***	−0.358
	(−2.46)	(−2.44)	(−0.92)	(−2.78)	(−2.77)	(−1.03)
Emotional Support		0.660 ***	2.527 ***		0.660 **	2.527 ***
		(3.01)	(2.97)		(4.04)	(3.63)
Responsiveness		0.295 ***	0.464 **		0.295 ***	0.464 **
		(5.42)	(2.19)		(6.19)	(2.52)
Service Continuity		0.386 ***	−0.363		0.386 ***	−0.363
		(3.73)	(−1.15)		(5.21)	(−1.30)
Price × Emotional Support			−0.949 **			−0.949 ***
			(−2.31)			(−2.73)
Price × Responsiveness			−0.083			−0.083
			(−0.87)			(−0.92)
Price × Service Continuity			0.390 **			0.390 ***
			(2.45)			(2.72)
Physician-fixed effects	Yes	Yes	Yes	Yes	Yes	Yes
Month-fixed effects	Yes	Yes	Yes	Yes	Yes	Yes
Observations	2955	2955	2955	3765	3765	3765
Log likelihood	−291.290	* −291.108	−291.080	−5828.442	−5782.355	−5775.260
Wald (LR) chi2	25.11	64.15	76.36	65,999.54	66,091.72	66,105.91
Prob > chi2	0.000	0.000	0.000	0.000	0.000	0.000
Pseudo R2	0.266	0.266	0.267	0.850	0.851	0.851

Notes: z statistics in parentheses; * *p* < 0.10, ** *p* < 0.05, *** *p* < 0.01.

**Table 7:** Results of the adoption model with disease-fixed effects.

Variable	(1)	(2)	(3)
Constant	1.143 ***	0.632 ***	0.356
	(4.72)	(2.87)	(1.53)
Recommendation	0.043	0.046	0.047
	(1.01)	(1.18)	(1.21)
Article	0.077 **	0.077 **	0.075 **
	(2.19)	(2.43)	(2.40)
Price	−0.157 *	−0.128	0.027
	(−1.75)	(−1.58)	(0.29)
Emotional Support		0.241 ***	0.486 ***
		(6.84)	(3.42)
Responsiveness		0.111 ***	0.204 ***
		(14.27)	(6.54)
Service Continuity		0.270 ***	0.248 ***
		(13.94)	(3.30)
Price × Emotional Support			−0.129 *
			(−1.76)
Price × Responsiveness			−0.052 ***
			(−3.08)
Price × Service Continuity			0.011
			(0.27)
Physician-fixed effects	Yes	Yes	Yes
Disease-fixed effects	Yes	Yes	Yes
Observations	3765	3765	3765
F	3.22	98.11	67.51
Prob > F	0.022	0.000	0.000
R-squared	0.949	0.959	0.959
Adjusted R-squared	0.924	0.938	0.938

Notes: *t* statistics in parentheses. * *p* < 0.10, ** *p* < 0.05, *** *p* < 0.01.

**Table 8:** Results of the usage model with disease-fixed effects.

Variables	(1)	(2)	(3)
Constant	1.076 **	−0.247	−0.607
	(2.47)	(−0.53)	(−0.94)
Gift	0.149 ***	0.129 ***	0.130 ***
	(5.10)	(4.48)	(4.51)
Price	−0.669 ***	−0.642 ***	−0.448
	(−3.01)	(−2.87)	(−1.37)
Emotional Support		0.397 ***	1.849 ***
		(2.94)	(3.39)
Responsiveness		0.373 ***	0.478 ***
		(9.01)	(3.22)
Service Continuity		0.215 ***	−0.247
		(3.59)	(−1.15)
Price × Emotional Support			−0.761 ***
			(−2.75)
Price × Responsiveness			−0.056
			(−0.75)
Price × Service Continuity			0.249 **
			(2.18)
Physician-fixed effects	Yes	Yes	Yes
Disease-fixed effects	Yes	Yes	Yes
Observations	2955	2955	2955
Log pseudolikelihood	−2183.014	−2176.576	−2176.067
Wald chi2	37.98	142.76	150.87
Prob > chi2	0.000	0.000	0.000
Pseudo R^2^	0.195	0.197	0.197

Notes: z statistics in parentheses; * *p* < 0.10, ** *p* < 0.05, *** *p* < 0.01.

## Data Availability

The data that support the findings of this study are available from the corresponding author upon reasonable request.

## Appendix A

**Table A1:** The labeling criteria and examples.

Criterion	Examples
Daily greetings	“Hello! Welcome to my online clinic.” “How’s it going these days?”
Positive comments on recovery outcomes	“Your situation has quite a bit of improvement” “You’re recovering well.”
Reassurance and encouragement	“Don’t worry!” “We’ll work together!”
Clarifications aimed at alleviating uncertainty regarding treatment modalities and recovery processes	“The success rate of this surgery is quite high” “It will improve over time.”

## Appendix B

**Table A2:** Descriptive statistics and correlations of variables.

Variable	Min	Max	Mean	Std.Dev.	1	2	3	4	5	6	7	8	9
1. Adoption	0	2600	34.302	90.806	1								
2. Usage	0	254	6.785	17.675	0.649 ***	1							
3. Emotional Support	0	13.333	0.714	0.808	0.375 ***	0.326 ***	1						
4. Responsiveness	1	3	2.600	0.684	0.474 ***	0.355 ***	0.442 ***	1					
5. Service Continuity	0	1	0.408	0.401	0.494 ***	0.286 ***	0.073 ***	0.231 ***	1				
6. Price	0	3000	138.560	194.704	0.249 ***	0.135 ***	0.133 ***	0.164 ***	−0.025	1			
7. Recommendation	0	5	3.974	0.461	0.679 ***	0.468 ***	0.192 ***	0.249 ***	0.379 ***	0.292 ***	1		
8. Article	0	155	0.479	5.163	0.196 ***	0.132 ***	0.081 ***	0.091 ***	0.098 ***	0.078 ***	0.199 ***	1	
9. Gift	0	151	1.152	4.218	0.611 ***	0.517 ***	0.234 ***	0.246 ***	0.219 ***	0.218 ***	0.530 ***	0.299 ***	1

Notes: * *p* < 0.10, ** *p* < 0.05, *** *p* < 0.01.
